# Atomically-precise Au_22_(Lys-Cys-Lys)_16_ nanoclusters for radiation sensitization

**DOI:** 10.1186/s12951-025-03256-7

**Published:** 2025-03-07

**Authors:** Elham Zeinizade, Goonay Yousefalizideh, Parimah Aminfar, Matthew Horn, Lili Ding, Layla Pires, Alina Jaglanian, Lucie Malbeteau, Kristen Harrington, Carla Calçada, Mohamad Dukuray, Brian C. Wilson, Marianne Koritzinsky, Juan Chen, Kevin G. Stamplecoskie, Gang Zheng

**Affiliations:** 1 Princess Margaret Cancer Centre, University Health Network, Toronto, ON M5G 1L7 Canada; 2 Department of Medical Biophysics, University of Toronto, Toronto, ON M5G 1L7 Canada; 3 Department of Chemistry, Queen’s University, Kingston, ON K7L 3N6 Canada; 4 Department of Radiation Oncology, University of Toronto, Toronto, ON Canada; 5 Institute of Medical Science, University of Toronto, Toronto, ON M5G 1L7 Canada

**Keywords:** Atomically precise, Gold nanoclusters, Radiosensitizer, Ultra small size, Biocompatibility, Renal clearance, Cancer radiation therapy

## Abstract

Radiotherapy is a leading method for cancer treatment, effectively eliminating cancer cells but often causing collateral damage to surrounding healthy tissue. Radiosensitizers aim to enhance the therapeutic effects of radiotherapy while minimizing harm to normal cells. We recently reported atomically-precise gold nanoclusters, Au_22_(Lys-Cys-Lys)_16_, synthesized *via* a photochemical method coupled with a novel accelerated size-focusing procedure. These nanoclusters exhibit a distinct luminescence emission profile, reflecting exceptional optical purity and the absence of contamination from other nanocluster species. They demonstrate efficient oxygen radicals generation under light irradiation. In this study, we comprehensively evaluated the radiosensitization potential of Au_22_(Lys–Cys–Lys)_16_ nanoclusters in vitro and in vivo, alongside their pharmacokinetics, biodistribution and toxicity. The nanoclusters demonstrated high stability under physiological conditions and efficient internalization in tumor cells, achieving dose enhancement factors of 2.0 and 1.6 in KB and 4T1 tumor cells, respectively, under 225 kVp X-ray irradiation. Mechanistic investigations revealed enhanced radiation-induced DNA damage and disruption of DNA repair pathways. The radiosensitizing effects were further validated in radioresistant pancreatic ductal adenocarcinoma cells using the clonogenic assay and γH2AX analysis of double-strand breaks, as well as in a duck chorioallantoic membrane model. With ultra small size (~ 1.7 nm) and favorable surface framework, the nanoclusters exhibited relevant pharmacokinetics (circulation half-life, t₁_/_₂ = 10.4 h) and renal clearance. In a KB tumor-bearing mouse model, Au_22_(Lys–Cys–Lys)_16_ significantly delayed tumor progression and prolonged survival under 8 Gy irradiation without observed side-effects. These findings establish Au_22_(Lys–Cys–Lys)_16_ nanoclusters as a potentially translatable radiosensitizer, advancing cancer radiotherapy strategies.

**Supplementary Information:**

The online version contains supplementary material available at 10.1186/s12951-025-03256-7.

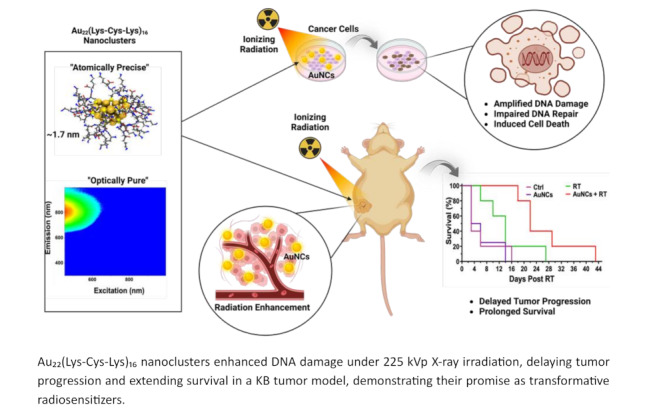

## Introduction

Radiotherapy is a primary modality in cancer treatment [1, 2], utilized in over 50% of patients either stand-alone or combined with other treatments such as surgery, chemotherapy or immunotherapy [3]. High-energy radiation effectively kills cancer cells but also poses significant risk to surrounding normal tissues [4]. Hence, the maximum tolerated dose of these tissues restricts the radiation doses that can be delivered to the tumor, thereby limiting the achievable efficacy despite technological advances that have improved the precision of radiation dose delivery. Additionally, radiation resistance of tumor is a major challenge in several cancer types [5], so that alternative approaches are required to maximize the radiation effect at the cellular level. Radiosensitizers that selectively and efficiently accumulate in tumor cells are one approach to widen the therapeutic window.

Over the past two decades, high-Z metallic materials have been investigated for this purpose by enhancing the local energy dose deposition [6]. Gold nanoparticles (Z = 79) (AuNPs) exhibit significantly higher radiation absorption than tissue (~ 100-fold enhancement in the keV energy range) [7], and are chemical stable, inert and biocompatible [8–11]. However, most AuNPs reported to date are relatively large (~ 15–100 nm), leading to potential entrapment by the reticuloendothelial system (RES) and resulting in low tumor uptake and high accumulation in liver and spleen [12]. Reducing the particle size should mitigate this effect [13]. Thus, for example, Hainfeld et al. demonstrated that 1.9 nm AuNPs are cleared primarily through the kidneys and can be efficiently and selectively delivered to tumor *via* intravenous administration, achieving high concentration and significant high-Z radiation dose enhancement [11]. In contrast to nanoparticles composed of multiple functional components with varied sizes and shapes, atomically precise nanoclusters possess a well-defined structure and chemical composition, facilitating accurate tracking within the body and investigation of potential translatable products.

Gold nanoclusters (AuNCs), composed of a few to tens of gold atoms, possess unique physicochemical properties arising from their discrete electronic structure. Unlike bulk gold, these nanoclusters have well-defined energy levels analogous to molecular orbitals, with distinct transitions from highest to lowest molecular orbitals that are specific to the size and composition of each cluster [14–16]. The two main types of transitions are metal-metal (core transitions) and metal-ligand (or ligand-metal) charge transfers, which are not observed in larger metal nanoparticles [17, 18]. This gives controllable functionality for diverse applications, including chemo/biosensing, imaging and photodynamic or photothermal therapies [19, 20]. Additionally, AuNCs hold promise as radiation sensitizers as the result of the high-Z dose-enhancement effect. Their molecular structures can be tailored to optimize biocompatibility, renal clearance and targeted delivery [21].

We have recently developed atomically-precise AuNCs, stabilized with a tri-amino acid peptide ligand, Lysine-Cysteine-Lysine (Lys–Cys–Lys). The synthesis involved photochemical reduction of Au(I)-thiolate ligand (Lys-Cys-Lys) complexes, followed by a novel light-activated size-focusing step [22]. These AuNCs consist of 22 gold atoms stabilized by 16 Lys–Cys–Lys ligands. This precise formulation allows tight control over size and shape, while ensuring high water solubility. Additionally, the clusters are “optically pure”, exhibiting a single luminescence emission peak at ~ 790 nm, independent of the excitation wavelength. The clusters also demonstrate excellent photodynamic activity by efficiently generating oxygen radicals [22]. Here, we investigated this material as a novel radiosensitizer.Our evaluation encompassed their stability under various physiological conditions, pharmacokinetics, biodistribution, tissue toxicity and radiation dose enhancement in tumor models in vitro, *ex ovo* and in vivo. We hypothesized that the atomically-precise nature of Au_22_(Lys-Cys-Lys)_16_ NCs, characterized by uniform molecular-level size and shape and by exceptional stability, would result in reproducible and predictable behavior in biological systems. Furthermore, we proposed that their efficient oxygen-radical generation and high atomic number would contribute to significant radiation dose enhancement.

## Results and discussion

### Au_22_(Lys-Cys-Lys)_16_ synthesis and characterization

**Fig. 1:**
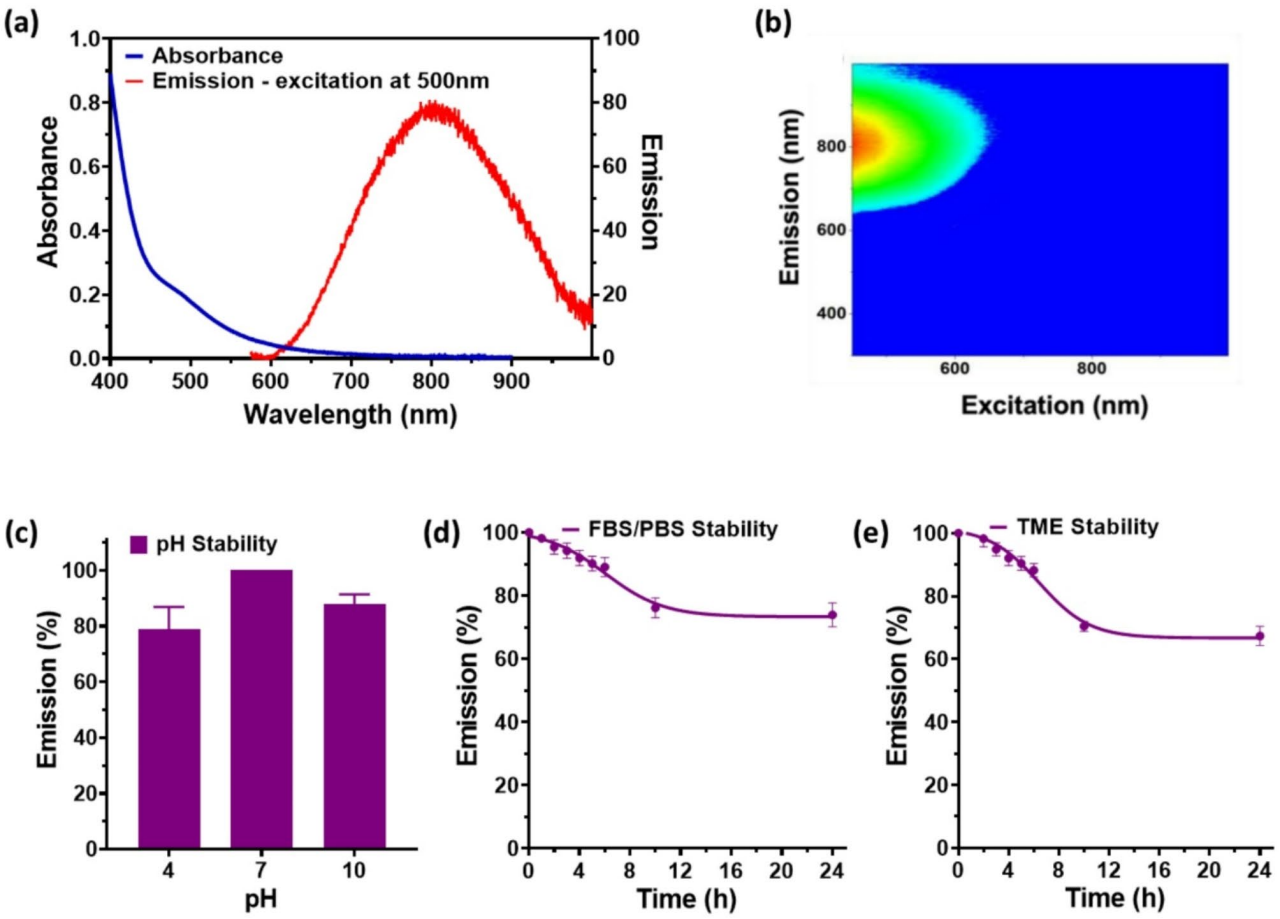
**(a)** Au_22_(Lys-Cys-Lys)_16_ nanocluster absorption and fluorescence emission spectra (𝝀exc = 500 nm). **(b)** Fluorescence EEM. **(c)** Emission under different pH, normalized to that for pH 7. Emission stability as a function of time in (**d**) 50% FBS in PBS and in **(e)** simulated TME (pH 6.5, 10 µM H_2_O_2_) normalized to that at time 0

In a previous study, we reported the use of fluorescence Excitation-Emission Matrix (EEM) spectroscopy coupled with Parallel-Factor (PARAFAC) spectral decomposition to monitor the formation of emissive intermediates during the synthesis of Au_22_(Lys–Cys–Lys)_16_ nanoclusters and to ensure quality control of the final product [22]. UV-Vis absorption and fluorescence spectroscopies (Fig. 1a) and EEM spectroscopy (Fig. 1b) were used to assess the stability of the AuNCs under physiological conditions. Fig. 1c, d, e show the optical stability of the AuNCs under varying pH condition, in the presence of serum, and within a tumor microenvironment (TME) model. As shown in Fig. 1c, the absorbance profile remained stable across a broad pH range. However, under both strongly acidic (pH 4) and basic (pH 10) conditions, a slight decrease in fluorescence intensity was observed, while the overall absorption and emission spectra remained largely unchanged (Figure S1a, b). The minor change in fluorescence intensity is likely attributed to variations in the protonation states of the Lys-Cys-Lys peptide ligand at different pH. The AuNCs stability in a serum-mimicking environment (50% fetal bovine serum (FBS) in phosphate buffered saline (PBS)) was evaluated through real-time monitoring of the absorption and fluorescence spectra. As shown in Fig. 1d and Figure S1c, d, only a gradual reduction in fluorescence was observed over time, with > 70% of the initial intensity retained after 24 h. The tumor microenvironment (TME) exhibits unique chemical conditions, including mild acidity and elevated hydrogen peroxide levels. To simulate these conditions in solution, the AuNCs were incubated in a buffer containing 10 µM H_2_O_2_ at pH 6.5. The absorption and emission spectra remained largely unchanged, with 70% of the initial fluorescence intensity retained over 24 h (Fig. 1e, Figure S1e & f). These data indicate that the AuNCs exhibit excellent stability under physiological conditions, supporting their potential for biomedical applications.

### Dark toxicity and intracellular uptake in cancer cells in vitro

**Fig. 2:**
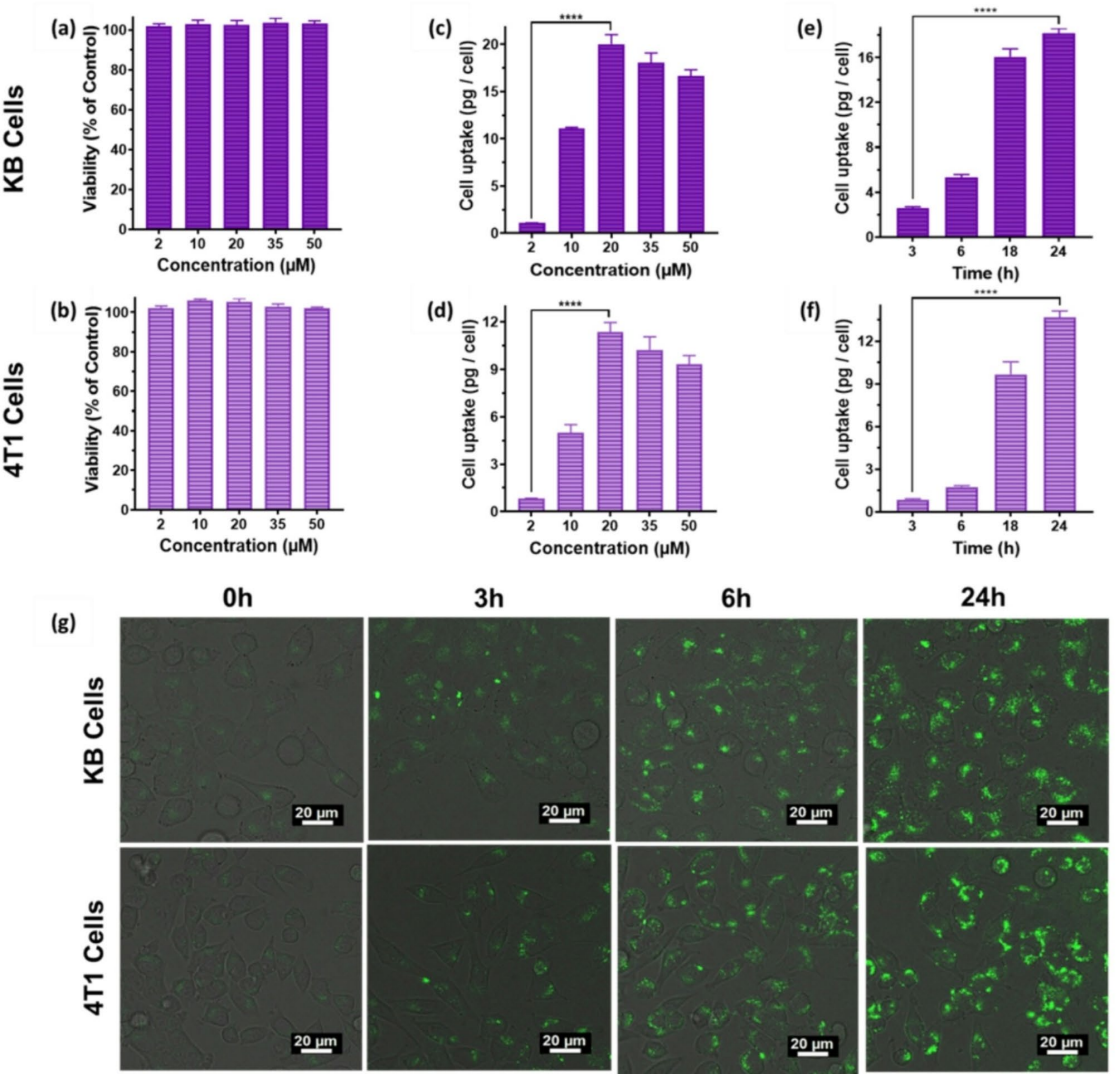
**(a**,** b)** Cell viability after 24 h incubation with AuNCs at different concentrations: error bars ± 1 s.d. (*n* = 3); **(c**,** d)** Cellular uptake measured by ICP-MS after 24 h incubation with different concentrations of AuNCs; **(e**,** f)** Cellular uptake with 20 µM of AuNCs at different time points (****P < 0.0001, ***P < 0.001, **P < 0.01, *P < 0.05). **(g)** Confocal fluorescence microscopy images demonstrating cellular internalization after incubation with 10 µM NCs for different times

The dark cytotoxicity of the AuNCs was evaluated using the AlamarBlue assay in two cancer cell lines, human-derived KB and murine breast cancer 4T1, over 24 h incubation at concentrations ranging from 2 to 50 µM. No significant cell death was observed (Fig. 2a, b). The intracellular uptake was measured by inductively coupled mass spectrometry (ICP-MS) after incubation at 2–50 µM for 24 h. The results demonstrated concentration-dependent increase in intracellular uptake in the range 2–20 µM for both cell lines, with > 10-fold enhanced uptake at 20 µM compared to 2 µM (*P* < 0.0001) (Fig. 2c, d). Above 20 µM the uptake reached saturation. Additionally, time-dependent increases in uptake were observed when cells were incubated with a fixed concentration of 20 µM for 1, 3, 6 and 24 h, with significantly higher accumulation at 24 h compared to 3 h (*P* < 0.0001) (Fig. 2e, f).

The intracellular concentration of gold was > 10 pg per cell after 24 h incubation with 20µM of AuNCs, significantly higher than reported values for other gold nanoclusters structured with cyclic peptide ligands, such as cyclic arginine-glycine-aspartic acid-D-tyrosine-cysteine (c(RGDyC)) (< 1pg per cell) [23] or Folate (FA) ligands (< 0.04 pg per cell) [24]. This high uptake is likely due to the positively-charged Lys-Cys-Lys tripeptide framework, which interacts favorably with the negatively-charged cell membrane [25]. The intrinsic fluorescence of Au_22_(Lys-Cys-Lys)_16_ NCs further enabled tracking of intracellular uptake by confocal microscopy, confirming efficient and time-dependent internalization (Fig. 2g).

### Efficacy of Au_22_(Lys-Cys-Lys)_16_ as a radiosensitizer

**Fig. 3:**
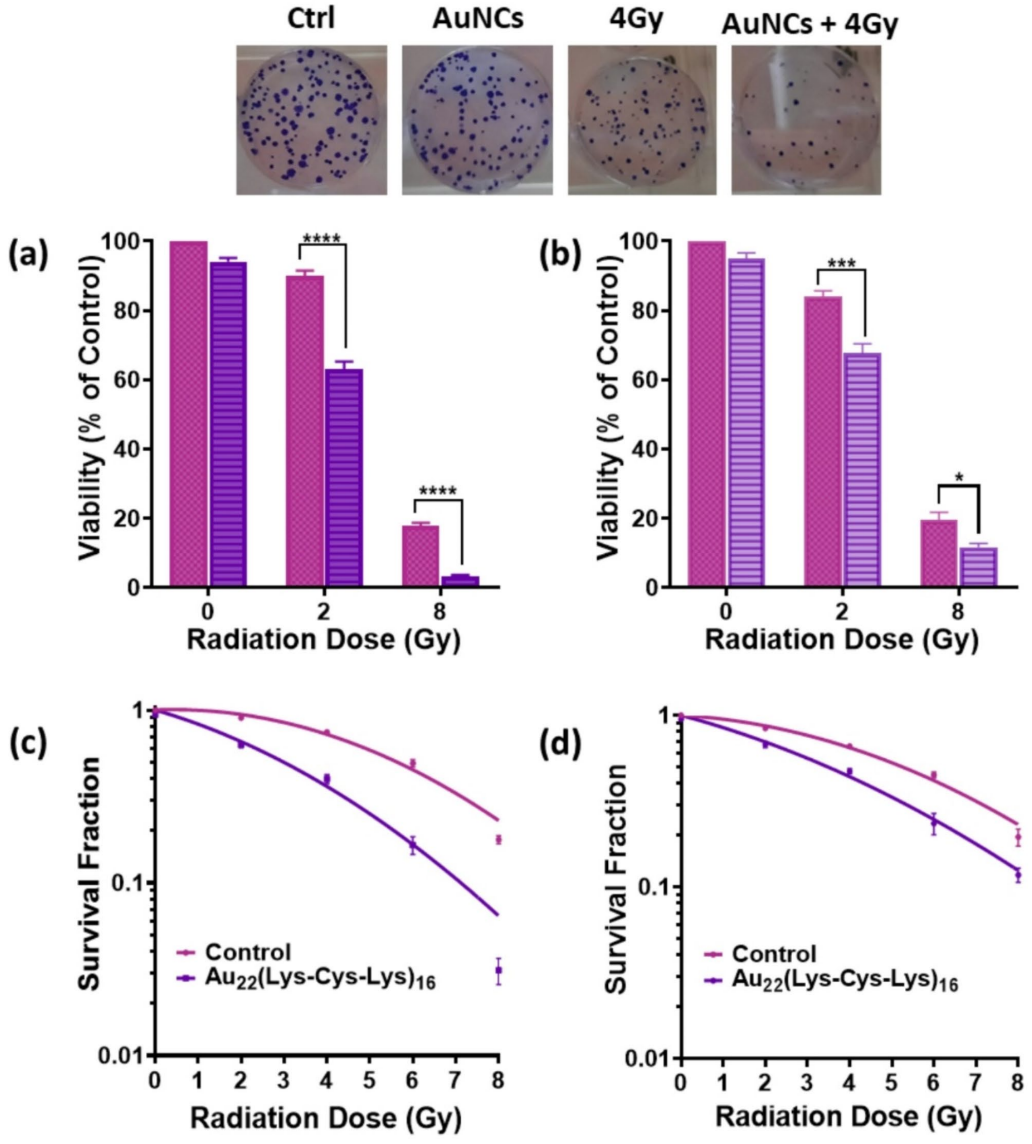
Cell viability of **(a)** KB and **(b)** 4T1 cells measured by colony formation as a function of radiation dose without (control) and with nanoclusters (20 µM, 24 h incubation). **(c**,** d)** Corresponding clonogenic cell survival curves: ****P < 0.0001, ***P < 0.001, **P < 0.01, *P < 0.05. The images at the top are examples of colony formation

Given that the core of the Au_22_(Lys-Cys-Lys)_16_ NCs is composed of multiple gold atoms capable of strong interactions with ionizing radiation, along with their favorable biocompatibility and efficient intracellular uptake, these nanoclusters are anticipated to serve as an effective radiosensitizer. Colony-forming assays were performed after 24 h incubation in vitro, followed by exposure to 2, 4, 6–8 Gy of 225 kVp X-rays. Treatment groups included no-treatment, AuNCs-only, radiation-only, and AuNCs plus radiation. Au_22_(Lys-Cys-Lys)_16_ NCs showed no significant cytotoxicity in the absence of X-radiation but enhanced cell death at all radiation doses in both cell lines (Fig. 3a, b). Thus, at 2 Gy the survival of KB and 4T1 cells was reduced to 63.0 ± 2.4% (*P* < 0.0001) and 67.6±2.8% (*P* < 0.001), respectively, compared to 87.2 ± 3.5% and 82.8 ± 3.3% with X-ray irradiation only. At 8 Gy, the AuNCs further reduced survival to 5.3 ± 1.4% (*P* < 0.0001) for KB cells and 11.7 ± 1.1% (*P* < 0.05) for 4T1 cells, compared to 17.8 ± 1.0% and 19.5 ± 2.2% for radiation alone.

The corresponding survival curves are presented in Fig. 3c and d. The dose enhancement factor (DEF) was calculated as the ratio of the dose for 50% survival without and with the AuNCs. The values were 2.0 ± 0.3 and 1.6 ± 0.1 for the KB and 4T1 cells, respectively. The difference may be partially attributed to the higher accumulation in KB cells (20 pg/cell) compared to 4T1 cells (11.4 pg/cell). Other gold nanoclusters, such as c(RADyC)-AuNCs [23], AuNC-ASON for combined radiation/gene therapy [26], peptide-based gold nanoclusters like CCYKFR–AuNCs and CCY–AuNCs [27], as well as AuNCs@His and GSH-AuNCs@His [28], have reported DEFs ranging from 1.21 to 1.8.

The radiosensitization effect of AuNPs and AuNCs is generally influenced by multiple factors, including size, shape, surface chemistry, intracellular concentration and radiation type [29–32]. For example, altering the concentration of AuNPs in HeLa cells yielded radiation enhancement factors of 1.27 and 1.44 [33]. Additionally, variation in nanocluster sizes and surface chemistry have shown significant effects on enhancement factors [34, 35]. The DEFs observed in this study underscore the importance of the ultra-small size (1.7 nm) and biocompatible tripeptide framework, which contribute to effective intracellular uptake and radiosensitization.

### Au_22_(Lys-Cys-Lys)_16_-mediated DNA damage and apoptotic cell death following radiation

**Fig. 4:**
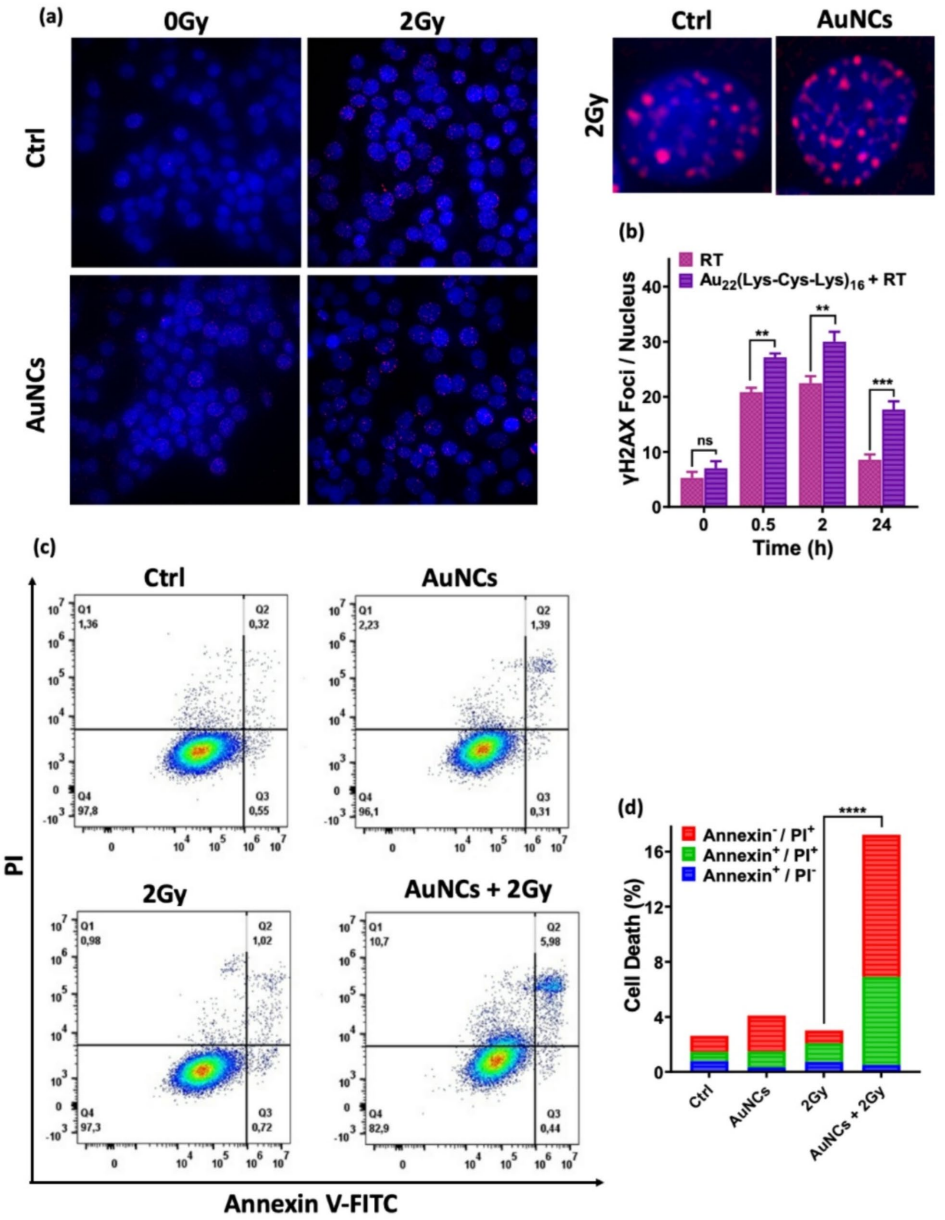
**(a)** Examples of **γ**H2AX fluorescence microscopy images in KB cells at 2 h following 2 Gy X-ray exposure without or with nanoclusters (10 µM, 2 h incubation). **(b)** Time-course of γH2AX foci per nucleus following X-ray irradiation **(c)** Flow cytometry of KB cell death modes at 24 h post treatment. **(d)** Corresponding histograms. ****P < 0.0001, ***P < 0.001, **P < 0.01, *P < 0.05

The radiation sensitization effect was further investigated by measuring DNA double-strand breaks (DSBs) using γH2AX staining. Apoptosis and necrosis were assessed using Annexin V-FITC apoptosis staining and propidium iodide (PI) staining, respectively. The reduction profiles of the γH2AX signal over time were also evaluated to monitor the DNA repair process [36]. KB cells were incubated with the 10 µM AuNCs for 24 h, followed by exposure to 2 Gy. The cells were then fixed and analyzed for γH2AX foci using immunofluorescence staining. As shown in Fig. 4a, cells treated with nanoclusters alone exhibited a low number of γH2AX foci per nucleus, similar to the untreated controls. In contrast, cells incubated with AuNCs and exposed to 2 Gy showed a marked increase in γH2AX foci after 2 h (30 ± 5) compared to radiation-only group (22 ± 4) (*P* < 0.01) (Fig. 4b). At 24 h post treatment, γH2AX levels decreased markedly in cells exposed to radiation alone, while elevated levels persisted in cells treated with AuNCs plus 2 Gy radiation, suggesting that the nanoclusters not only amplify the initial DNA damage but also prolong impairment of DNA repair. When DNA damage remains unrepaired, cells are more likely to undergo apoptosis or other forms of cell death. Apoptosis (Annexin V positive) and necrosis (PI positive) were assessed by flow cytometry using a commercial assay. As shown in Fig. 4c, neither X-ray treatment alone nor AuNCs alone resulted in significant increase in cell death (2.7 ± 1.7% and 3.9 ± 1.3%, respectively) compared to the untreated control cells (2.2 ± 1.1%). However, cells incubated with AuNCs followed by 2 Gy radiation exhibited a marked increase in cell death (17.1 ± 2.4%: *p* < 0.0001 compared with controls) (Fig. 4d). This is notably higher than values reported for other Au nanoclusters, such as GSH-AuNCs@His (4.9–8.8% for 6 Gy) [28] and GNS-FA (4.1–10.7% for 6 Gy) [37].

In summary, these findings indicate that these nanoclusters markedly amplify X-ray-induced DNA damage, trigger apoptosis and necrosis, thereby enhancing the efficacy of radiation therapy.

### Au_22_(Lys-Cys-Lys)_16_-mediated radiosensitization in radioresistant cancer cells

**Fig. 5:**
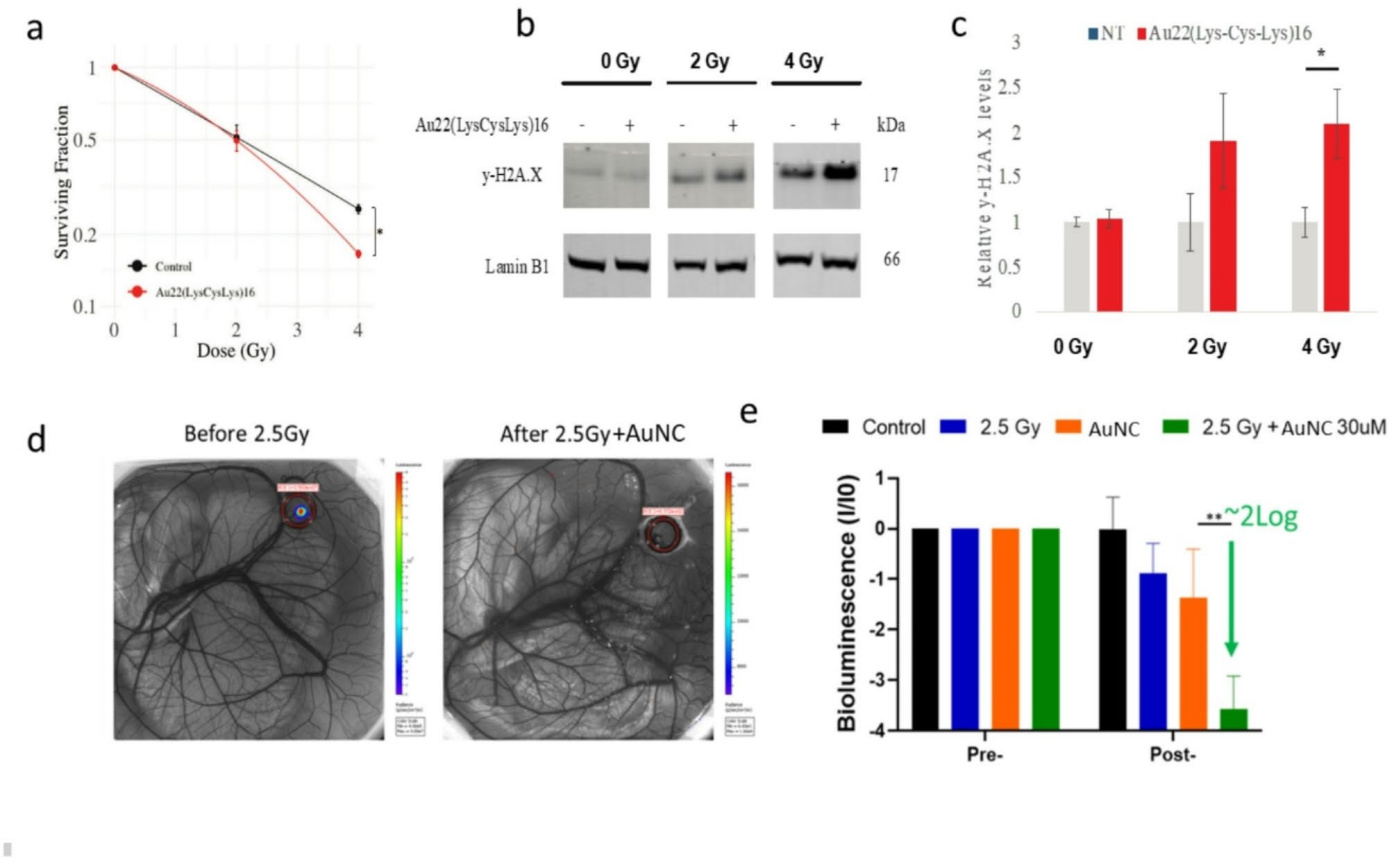
(**a**) Clonogenic survival of PANC-1 cells treated without (control) and with AuNCs (10 µM, 24 h incubation), normalized to non-irradiated controls. (**b**) Corresponding Western blot analysis performed 30 min post-treatment. (**c**) Quantification of γH2AX protein levels from panel (**b**). (**d**) Representative bioluminescence images of Panc-1-Luc-2 cells in the CAM model, comparing the AuNC-treated group (10µM, 24 h incubation) before and 24 h after 2.5 Gy irradiation. **(e**) Quantitative analysis of total BLI in the CAM model (*N* = 3/group. ****P < 0.001, ***P < 0.005, **P < 0.01, *P < 0.05)

As a model for intrinsic radiation resistance, the human-derived pancreatic ductal adenocarcinoma cell line PANC-1 was used. As shown in Figure S2, the AuNCs demonstrated concentration- and time-dependent uptake, with intracellular gold content exceeding 35 pg per cell after 48 h incubation at 10 µM. Clonogenic survival (Fig. 5a) was minimally affected by the AuNCs alone but there was a marked radiosensitization effect at 4 Gy, along with corresponding radiation dose-dependent increase in double-strand breaks (Fig. 5b, c).

To further evaluate the radiosensitizing potential of the AuNCs, tumors grown on a duck chorioallantoic membrane (CAM) that simulates the tumor microenvironment and vascular network were used as a rapid and cost-efficient intermediate platform between in vitro and fully in vivo models [38]. PANC-1-Luc-2 cells, which express luciferase, were utilized to enable longitudinal bioluminescence imaging (BLI) for monitoring tumor growth and treatment response. As shown in Fig. 5d, there was a significant reduction in the bioluminescence signal at 24 h post-treatment in the AuNC plus 2.5 Gy group compared to the X-ray-only control.

### In vivo behavior of Au_22_(Lys-Cys-Lys)_16_ under systemic administration

**Fig. 6:**
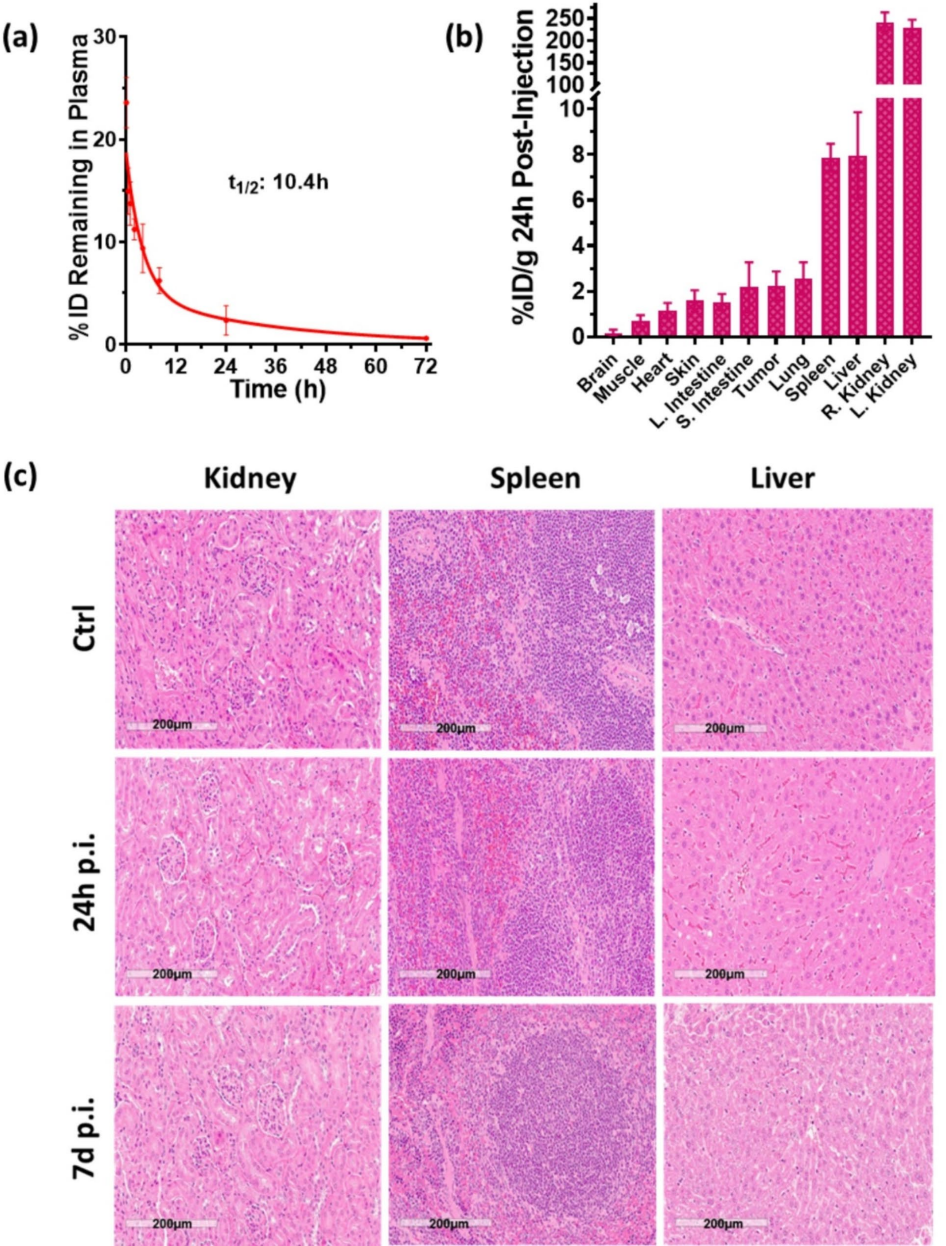
**(a)** Plasma clearance post *i.v.* injection of 10 mg/kg AuNCs in healthy BALB/c mice (*n*= 5). **(b)** Biodistribution in tumor and normal tissues collected 24 h post *i.v.* injection. **(c)** Examples of H&E-stained tissue sections at 24 h and 7 d post-*i.v.* injection

The pharmacokinetics of the AuNCs were first evaluated in healthy mice following tail-vein injection of 10 mg/kg, with blood sampling from 5 min to 72 h post-injection. The gold concentration in blood over time was fitted to a single-exponential decay model (Fig. 6a), yielding an elimination half-life (t_1/2_) of 10.4 ± 0.9 h. This is significantly longer than for most reported gold nanoclusters, such as cyclic RGD peptide coated c(RGDyC)-AuNCs (~ 2 h) [23], bovine serum albumin-coated BSA-AuNCs (~ 0.25 h) and glutathione coated GSH-AuNCs (~ 0.75 h) [35]. It is also longer than quaternary ammonium-capped QA-AuNCs (~ 7.5 h) [39], and comparable to PEGylated gold nanoparticles, PEG-AuNPs (~ 9 h) [40]. This prolonged circulation time could potentially enhance tumor accumulation, thereby improving therapeutic efficacy.

Efficient clearance of the nanoclusters from normal tissues is important to minimize toxicities. Clearance is typically governed by renal or hepatic pathways, depending on particle size and surface properties, with renal clearance being more favorable due to faster kinetics [41]. Nanoparticles with a hydrodynamic diameter < ~ 5 nm are generally efficiently filtered by the kidneys [42, 43]. The hydrodynamic size of nanoclusters is influenced by both the gold core and the surface ligands [44]. For example, Au_25_ nanoclusters coated with BSA have a hydrodynamic diameter of 7 nm and tend to accumulate in the liver and spleen. In contrast, glutathione (GSH)-protected Au_25_ nanoclusters with a 2 nm hydrodynamic diameter exhibit significant urinary clearance [45], underscoring the critical role of surface ligands.

The biodistribution of the AuNCs was assessed 24 h post intravenous (*i.v.*) injection of 10 mg/kg in athymic nude mice bearing subcutaneous KB tumors. As shown in Fig. 6b, tumor accumulation was 2.4 ± 0.5% of the injected dose per gram (ID/g), while the kidney showed the highest accumulation (> 200% ID/g), compared with ~ 8% ID/g in the liver and spleen. The kidney-to-blood, liver-to-blood and spleen-to-blood ratios were 11.0 ± 2.1, 2.9 ± 0.4 and 0.2 ± 0.1, respectively. Urine samples collected at various intervals post-injection, measured for gold content by ICP-MS, showed a progressive increase in excretion up to 24 h after administration, followed by a gradual decrease. The total excreted gold content continued to rise over the 72 h experimental period (Figure S3).

To further assess potential toxicity, body weight and pathology of major organs were examined 24 h to 7 d post *i.v.* injection of 10 mg/kg AuNCs. No significant weight loss, abnormal clinical signs or behavioral changes were observed. H&E tissue staining of kidney, liver and spleen revealed no pathological changes at either time point (Fig. 6c).

In summary, the favorable solubility, prolonged circulation time, preferential renal clearance and minimal systemic toxicity highlight the potential of Au_22_(Lys-Cys-Lys)_16_ nanoclusters for in vivo clinical applications. These findings align with prior studies showing that surface modification of nanoparticles with naturally-occurring biomolecules and ligands [46–48] can enhance renal filtration while reducing accumulation in the liver and spleen [49].

### Radiosensitization of tumor in vivo

**Fig. 7:**
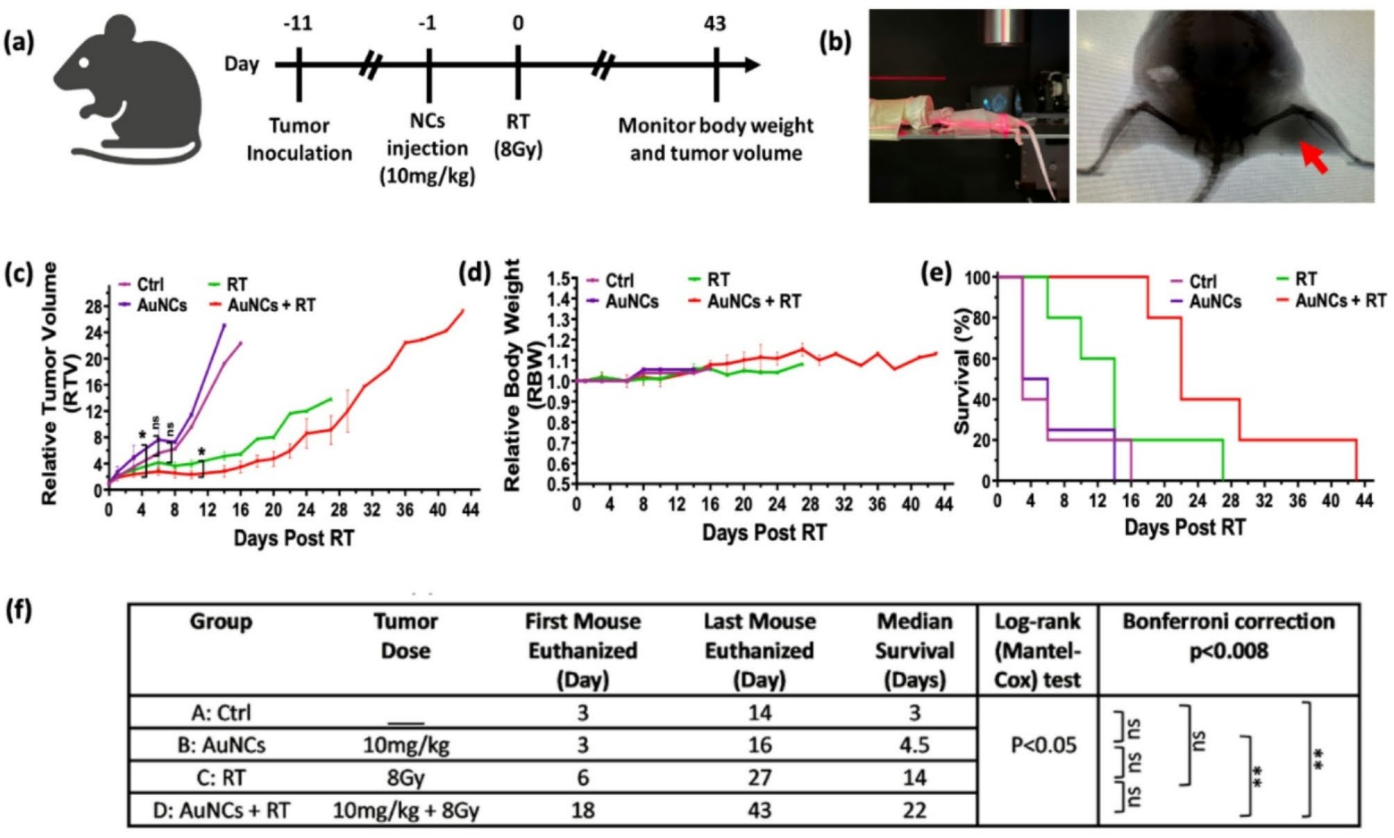
In vivo AuNC-enhanced radiotherapy in a subcutaneous KB tumor mouse model. **(a)** Schematic of the experimental timelines. **(b)** Irradiation setup and X-ray CT scan showing the tumor (arrow). Time-course post irradiation (means ± 1 s.d.: *n* = 5 per group) of **(c)** tumor volume normalised to the pre-treatment value, **(d)** normalised body weight and **(e)** survival profile for each treatment group. **(f)** Treatment information for each group and response analysis are summarized in the table. For survival statistical analysis, the log-rank (Mantel-Cox) test was used to compare survival differences across 4 groups. To account for multiple comparisons across 4 pairs, the significance threshold was adjusted using the Bonferroni correction. ‘ns’ indicates no statistical significance, while ‘**’ denotes a *p*-value < 0.01

Fig. 7a outlines the in vivo experimental protocol, where mice received 10 mg/kg of AuNCs *via* intratumoral (IT) injection, followed 24 h later by 8 Gy irradiation. Fig. 7b shows the irradiation setup and X-ray CT imaging of the tumor region. IT injection was chosen for this proof-of-principle study to ensure precise control over the AuNC concentration. Body weight and tumor volume were monitored 3 times weekly over 6 weeks using digital calipers. Fig. 7c displays that the no-treatment and AuNCs-only controls showed rapid tumor growth, whereas radiation-alone and radiation plus AuNCs significantly inhibited tumor growth, with the combination treatment achieving the greatest growth delay. There were no significant differences in body weight among the groups (Fig. 7d). Survival curves (Fig. 7e) showed that the untreated and AuNCs-only controls began reaching the study endpoint (tumor volume of 1500 mm^3^) by day 3, while this endpoint was not reached until day 6 for the X-ray only group and until Day 18 for the combined treatment group. By days 14, 16 and 27, all mice in the first 3 groups had reached the endpoint, whereas the combination treatment group exhibited 100% survival at day 16 and 40% survival at day 27, continuing until day 43 (Fig. 7e). The median survival time for the combined treatment group was 22d, significantly longer than the 3-14d in the 3 control groups (Fig. 7f). Additionally, as shown in Fig. 7d, body weight increased slightly across all groups during the treatment period, indicating good tolerability of the treatment. These results suggest that intratumor accumulation of Au_22_(Lys-Cys-Lys)_16_ nanoclusters significantly enhances the efficacy of radiotherapy, leading to delayed tumor growth and improved survival in the KB tumor model.

## Conclusions

The goal of radiosensitizers is to enhance preferentially the radiotherapy response in tumor while minimizing harm to healthy tissues. Here, we explored the radiosensitization potential of novel, atomically-precise gold nanoclusters stabilized by Lys–Cys–Lys tripeptides. Mechanistic investigations revealed that, under X-ray irradiation, the AuNCs increased DNA damage and disrupted DNA repair in both human-derived KB cells and murine-derived 4T1 cancer cells, resulting in dose-enhancement factors of 2.0 ± 0.3 and 1.6 ± 0.1, respectively. The effect was likely facilitated by efficient internalization driven by the surface peptide (Lys-Cys-Lys) framework. AuNCs-mediated radiosensitization was further demonstrated in radioresistant PDAC cells in vitro using clonogenic survival assays and γH2AX phosphorylation analysis, as well as in an *ex ovo* CAM model. Additionally, the nanoclusters exhibited stable photophysical properties across the physiological pH range (6–10) and excellent biocompatibility. Their small size (~ 1.7 nm) and favorable surface structure facilitated prolonged blood circulation (t_1/2_ = 10.4 ± 0.9 h), enhancing tumor accumulation and predominantly renal clearance. This reduced the risk of long-term gold retention and potential associated toxicity. In vivo studies in KB tumor-bearing mice demonstrated significantly improved treatment outcomes, including delayed tumor growth and extended survival. The AuNCs showed tumor accumulation of 2.4 ± 0.5 ID%/g following *i.v.* administration. This accumulation could potentially be increased through molecular modifications, such as PEGlation and conjugation with targeted ligand, to enhance tumor-specific targeting and reduce off-target effect. Furthermore, the nanoclusters could be applied in standard hyperfractioned radiotherapy regime to minimize normal tissue damage and/or increase tumor response. Complementary investigations into the potential of these novel AuNCs for X-ray-activated photodynamic therapy (radiodynamic therapy) [50, 51] are on-going.

## Materials and methods

### Au_22_(Lys-Cys-Lys)_16_ NC synthesis

The Au_22_(Lys-Cys-Lys)_16_ nanoclusters were synthesized using photochemical reduction followed by size-focusing, as reported previously [22]. Briefly, a 9 mM aqueous solution of Lys-Cys-Lys (Can Peptide Inc, Canada) was reacted with 3 mM aqueous HAuCl_4_ using 9 mM Omnirad 2959 (IGM Resins, USA) as a photo-initiator, with the pH adjusted to 11 using 1 mM NaOH. The solution was purged with nitrogen gas for 15 min to remove oxygen, irradiated with UVA lamps (250 W/m^2^ for 12 min), and monitored for absorbance and fluorescence EEM spectra. After 12 min, a mixture of Au_x_(Lys-Cys-Lys)_y_ nanoclusters formed, exhibiting an absorbance feature around 600 nm and emission around 760 nm. To accelerate size focusing, the solution was further irradiated with a 620 nm LED (1.5 mW/cm^2^ for 24 h), monitoring the absorption spectrum (Fig. 1). After 24 h, stable Au_22_(Lys-Cys-Lys)_16_ NCs were identified, characterized by an absorbance peak at 500 nm and strong emission at 790 nm. These clusters were concentrated, purified using centrifugal filters with a 3 kDa cut-off, and stored at 4 °C, remaining stable for over one month. The absorbance spectra were recorded using a Cary 60 UV-Vis spectrometer (Agilent, USA), and EEM scans were obtained with a Duetta Fluorescence and Absorbance spectrometer (Horiba, USA), with Rayleigh scattering corrections applied using EzSpec™ software (Horiba, USA).

### Cell culture

Human KB epithelial carcinoma cells (a subline of HeLa cells), 4T1 mouse mammary carcinoma cells, and human pancreatic ductal adenocarcinoma PANC-1 cells were cultured in Roswell Park Memorial Institute (RPMI) 1640 medium (Gibco, USA). PANC-1-Luc-2 cells, a genetically engineered variant of PANC-1 expressing luciferase [52], were cultured in low-glucose Dulbecco’s Modified Eagle’s Medium (DMEM, Gibco, USA). All media were supplemented with 10% fetal bovine serum (FBS) and 1% penicillin/ streptomycin. Cells were incubated at 37 °C in 5% CO_2_/95% air.

### Cytotoxicity evaluation

Triplicates of cells were seeded in 24-well plates (2 × 10^4^ cells/well) for 48 h. The medium was then aspirated and medium containing different concentrations of the Au_22_(Lys-Cys-Lys)_16_ NCs was added. The cells were incubated for 24 h at 37 °C under 5% CO_2_/95% air, washed with PBS, and 1 mL AlamarBlue (1X) at a final concentration of 50 µg/mL was added to each well. Subsequently, the cells were incubated at 37 °C in the dark under a humidified atmosphere of 5% CO_2_ for 2 h. Fluorescence was measured using a microplate reader (CLARIOstar: BMG LABTECH, Germany) with excitation/emission at 530/590 nm.

### Quantification of cellular uptake

Cells were seeded in 24-well plates (2 × 10^4^ cells/well). After 48 h, the medium was aspirated and fresh medium containing different concentrations of AuNCs was added equally to each well. The cells were incubated for 3, 6, 18–24 h at 37 °C under 5% CO_2_/95% air, washed with PBS and detached using trypsin at 37 °C for 3 min. The cell suspensions were collected, counted, transferred into screw-top microcentrifuge tubes and centrifuged at 5,000 rpm for 5 min. The supernatant was removed and the cell pellets were dried completely using a speed vacuum system. The dried cells were digested in a 3:1 mixture of hydrochloric acid (37% w/v) and nitric acid (70% w/v) for 4 h at 60 °C while shaking at 300 rpm. After digestion, 1 mL double-distilled water (ddH_2_O) was added to each tube, centrifuged and diluted further with a mixed buffer containing 2% HNO_3_ and 2% HCl. Samples were analyzed using inductively coupled plasma mass spectrometry (ICP-MS) (NexION350Q: PerkinElmer, USA) with the mass analyzer set to detect elemental Au-196. A 500µL injection loop was used and each sample was mixed with carrier solution and iridium internal standard (20 µg/L) before injection.

### Confocal laser-scanning microscopy

Cells were seeded on Lab-Tek^®^II 8 well chamber cover glasses overnight. Medium containing the nanoclusters at a concentration of 10 µM was added to each well and incubated for 3, 6 and 24 h. The cells were then washed with PBS and the medium was replaced with fresh medium. The AuNC uptake was visualized using a confocal fluorescence microscope (SuperRes: Leica, Germany) with excitation/emission at 405 nm/600–800 nm.

### Clonogenic cell survival assay

Cells were seeded in 24-well plates (2 × 10^4^ cells/well) and incubated for 48 h at 37 °C under 5% CO_2_/95% air. The medium was aspirated, and fresh medium containing the AuNCs was added. Cells were incubated at 37 °C with 5% CO_2_/95% air for 24 h, then washed with PBS. KB and 4T1 cells were irradiated at 225 kVp (SmART-ATP Research Irradiator: Precision X-Ray, USA) at a dose rate of 4.6 Gy/min. Both X-ray and gamma irradiation are classified as low-LET (linear energy transfer) radiation, primarily generating reactive oxygen species that induce DNA damage, including single-strand breaks. To further investigate these effects, PANC-1 cells were irradiated with 661.7 keV gamma rays from a cesium-137 irradiator (Gammacell^®^ 40 Exactor: Nordion Inc, ON, Canada) at a dose rate of 2.7 Gy/min. After irradiation, the cells were rested overnight in the incubator and then re-plated in triplicate at low density in fresh cell culture media. After 10–12 d the colonies were fixed with cold methanol, stained with 0.01%w/v crystal violet solution, washed and colonies containing at least 50 cells were counted. The surviving fraction (SF) was calculated as the ratio of the plating efficiency (PE) of treated cells to that of untreated control cells. The dose enhancement factor (DEF) was subsequently determined by comparing the radiation dose required to achieve 50% survival in untreated cells with that required in treated cells.

### γH2AX immunofluorescence staining

The cells were incubated with the AuNCs at a concentration of 10 µM for 24 h, followed by 2 Gy X-irradiation. They were then fixed with 4% paraformaldehyde for 30 min and permeabilized with 0.5% Triton X-100 for 15 min. After washing with PBS, a blocking buffer (3% BSA in PBST solution) was added for 1 h. The cells were subsequently incubated with an anti-phospho-histone H2AX primary antibody (NovusBio, USA) overnight at 4 °C, followed by incubation with an Alexa Fluor 555 labeled-anti-mouse secondary antibody for 1 h at room temperature. To visualize the cell nuclei, the cells were stained with DAPI. DNA damage was evaluated using fluorescent imaging with appropriate filters. Images containing both DAPI-stained nuclei and γH2AX foci were captured. ImageJ software was used to set a threshold for foci and perform automated counting.

### Cell apoptosis

The cells were cultured in 6-well plates (4 × 10^4^ cells/well) for 48 h and then incubated with the AuNCs for 24 h, followed by 2 Gy irradiation. After an additional 24 h of incubation, the cells were detached from the surface using trypsin at 37 °C for 3 min, transferred to flow cytometry tubes and prepared according to the manufacturer’s instructions (eBioscience™ Annexin V Apoptosis Detection Kit FITC; Invitrogen, CA, USA).

### Immunoblotting

Protein was extracted using radioimmunoprecipitation assay (RIPA) lysis buffer supplemented with a protease and phosphatase inhibitor cocktail (Thermo Fisher Scientific; IL, USA) and 5µM ethylenediaminetetraacetic acid (EDTA). Protein quantification was performed using the bicinchoninic acid method (Pierce BCA Protein Assay Kit: Thermo Fisher Scientific, IL, USA) and measured on a plate reader (OMEGA: BMG Labtech, Germany) at 562 nm. Proteins were resolved by SDS–polyacrylamide gel electrophoresis, transferred onto a polyvinylidene difluoride (PVDF) membrane, and incubated with Anti-phospho-Histone H2A.X (Ser139) antibody, clone JBW301 (Sigma Aldrich, CA, USA) overnight at 4 °C. Goat-anti-mouse fluorophore-conjugated secondary antibodies (Li-COR Biosciences, NE, USA) was applied for 1 h at room temperature. Proteins were visualized using a near-infrared imaging system (Odyssey CLx: Li-COR Biosciences, NE, USA).

### Duck Chorioallantoic membrane (CAM) model

Duck-fertilized eggs were cleaned with 70% alcohol and incubated in a rotating incubator at 37°C with 40–60% humidity for 4 days. The eggs were then carefully cracked and the contents were transferred into sterile 10 × 10 cm weighing boats, covered with plastic lids and maintained in a stationary incubator under the same conditions. On day 10 post-fertilization, tumors were induced. For this, PANC-1-Luc-2 cells were trypsinized, centrifuged and gently mixed with Matrigel in a 3:1 volume ratio. To improve cell attachment, the CAM surface was scratched using a sterile paper tip before applying the cell mixture. A 1000uL pipette tip, previously cut to achieve an opening of approximately 3–4 mm, was used to dispense the cell and Matrigel mix (~ 10^7^ cells) carefully onto the CAM surface, avoiding large blood vessels. Four days later an 8 mm diameter silicon ring was placed around the tumor to facilitate drug administration, treatment delivery and tumor monitoring.

### *Ex-ovo* irradiation

Ten microliters of nanoclusters at a concentration of 10 μM were added to tumor cohorts (n > 10 per group). Twenty-four hours later, the tumors were irradiated with 225 kVP X-rays at a dose of 2.5 Gy. A 10 mm collimator and 0.3 mm thick Cu filter were used to focus X-ray beam on the tumor and minimize off-target exposure.

### Bioluminescence imaging (BLI) in the CAM model to track tumor growth

To monitor tumor response, 10 µl of luciferin were added to the silicon ring and after 30 s the bioluminescence ware imaged (Xenogen IVIS system: PerkinElmer, USA). The exposure time was adjusted according to tumor activity to reach counts greater than 8,000. Imaging was then repeated 24 h post-treatment.

### In vivo pharmacokinetics

All animal studies were carried with institutional approval (AUP#6880, University Health Network, Toronto, Canada). Nanoclusters (10 mg/kg) were injected into BALB/c mice (*n* = 5) *via* the lateral tail vein. Blood samples were collected before injection and at 5 min, 30 min, 1, 2, 4, 8, 24, 48 and 72 h post-injection, then centrifuged at 1000 rpm for 5 min. The resulting plasma samples were digested in a 3:1 mixture of hydrochloric acid (37% w/v) and nitric acid (70% w/v) for 4 h at 60 °C while shaking at 300 rpm. After digestion, 1 mL of ddH_2_O was added to each tube, centrifuged and diluted with a water buffer containing 2% HNO_3_ and 2% HCl. Gold content was measured using ICP-MS. The circulation half-life of the nanoclusters was calculated by fitting the data to a single-exponential decay curve (GraphPad Prism: USA).

### Biodistribution in vivo

AuNCs at a dose of 10 mg/kg were injected *via* tail vein into athymic nude mice (Envigo, USA) bearing subcutaneous KB tumors (5–6 mm diameter) on the right flank (*n* = 6). After 24 h, the mice were euthanized using CO₂ inhalation followed by cervical dislocation. Tumors and major organs, including muscle, skin, heart, kidneys, liver, lungs, spleen, small intestine, large intestine and brain, were carefully excised, weighed and dried using a speed vacuum. The dried samples were then digested in a 3:1 mixture of hydrochloric acid (37% w/v) and nitric acid (70% w/v) for 24 h. Subsequently, they were diluted with a water buffer containing 2% HNO_3_ and 2% HCl, and the gold content was quantified using ICP-MS.

### Renal excretion

AuNCs were injected into BALB/c mice (*n* = 5) *via* the lateral tail vein. Urine samples were collected prior to injection and at 8, 24, 48 and 72 h post injection. The samples were dried and digested in a 3:1 mixture of hydrochloric acid (37% w/v) and nitric acid (70% w/v) for 4 h at 60 °C while shaking at 300 rpm. The processed samples were analyzed by ICP-MS to quantify the excreted gold content.

### In vivo irradiations

Athymic nude mice (adult, female, ~ 23.5 g) were anesthetized with a 2% isoflurane and oxygen mixture. KB cells (2 × 10^6^) were inoculated subcutaneously into the right flank. The mice were monitored 3 times weekly until tumors reached 6–7 mm diameter. The mice were then divided randomly into 4 groups (*n* = 5 per group): untreated, radiation-only, AuNCs-only, AuNCs plus radiation. For the NC cohorts, AuNCs were injected intratumorally at a concentration of 5 mg/mL (50 µl) at a dose of 10 mg/kg using a 28 G needle. For the irradiated groups, mice were anesthetized with 2% isoflurane and oxygen, secured in a prone position, and tumors were irradiated with 225KVp X-rays at a dose rate of 4.6 Gy/min for 105 s, delivering a total dose of 8 Gy. Body weight and tumor volume were measured using digital calipers 3 times weekly for ~ 6 weeks until the study endpoint. Humane endpoint was defined as a tumor volume > 1500 mm^3^ or the presence of severe ulceration. The tumor volume was calculated using the formula: *V* = 0.5 × *L* (length) × *W*^2^ (width).

### Statistical analysis

Data were analyzed using commercial statistical software (Version 2023.9.1.494: R-Studio, Boston, MA, USA) with one-way ANOVA followed by Tukey’s HSD post-hoc test. Significance levels are indicated as follows: ****P < 0.0001, ***P < 0.001, **P < 0.01, and *P < 0.05.

## Electronic supplementary material

Below is the link to the electronic supplementary material.

Supplementary Material 1

## Data Availability

Data is provided within the manuscript or supplementary information files.

## Abbreviations

RES: Reticuloendothelial system
Au: Gold element
AuNCs: Gold nanoclusters
AuNPs: Gold nanoparticles
Lys-Cys-Lys: Lysine-Cysteine-Lysine
UV-Vis: Ultraviolet–Visible
EMM: Excitation-Emission Matrix
TME: Tumor microenvironment
FBS: Fetal bovine serum
PBS: Phosphate buffered saline
DEF: Dose enhancement factor
γH2AX: Gamma-H2A histone family member X (a Phosphorylated histone H2AX)
ICP-MS: Inductively coupled mass spectrometry
DNA: Deoxyribonucleic acid
DSBs: DNA double-strand breaks
i.v. injection: Intravenous injection
H&E: Hematoxylin and eosin
PI: Propidium iodide
CAM: Chorioallantoic membrane
BLI: bioluminescence imaging
