# Supplementary material for: Atomically-precise Au22(Lys-Cys-Lys)16 nanoclusters for radiation sensitization
Source: J Nanobiotechnology. 2025 Mar 7;23:185. doi: 10.1186/s12951-025-03256-7 (PMC11887354; doi:10.1186/s12951-025-03256-7)
Supplement: Supplementary file 1 — Supplementary Material 1 [file 12951_2025_3256_MOESM1_ESM.pdf]

Supporting information

Atomically-Precise Au_{22}(Lys-Cys-Lys)$_{16}$ Nanoclusters for Radiation Sensitization

Elham Zeinizade,[a,b] *Goonay Yousefalizideh,*[c] *Parimah Aminfar,*[c] *Matthew Horn,*[a] *Lili Ding,*[a] *Layla Pires,*[a] *Alina Jaglanian,*[a] *Lucie Malbeteau,*[a] *Kristen Harrington,*[c] *Carla Calçada,*[a] *Mohamad Dukuray,*[a] *Brian C. Wilson,*[a,b] *Marianne Koritzinsky,*[a,b,d,e] *Juan Chen,*[a*] *Kevin G. Stamplecoskie,*[c*] *Gang Zheng* [a,b,e*]

[a] Princess Margaret Cancer Centre, University Health Network, Toronto, Ontario, Canada, M5G 1L7

[b] Department of Medical Biophysics, University of Toronto, Toronto, Ontario, Canada, M5G 1L7

[c] Department of Chemistry, Queen's University, Kingston, Ontario K7L 3N6, Canada

[d] Department of Radiation Oncology, University of Toronto, ON, Canada

[e] Institute of Medical Science, University of Toronto, Toronto, Ontario, M5G 1L7

*Corresponding Author: juan.chen@uhn.ca, kevin.stamplecoskie@queensu.ca, gang.zheng@uhn.ca.

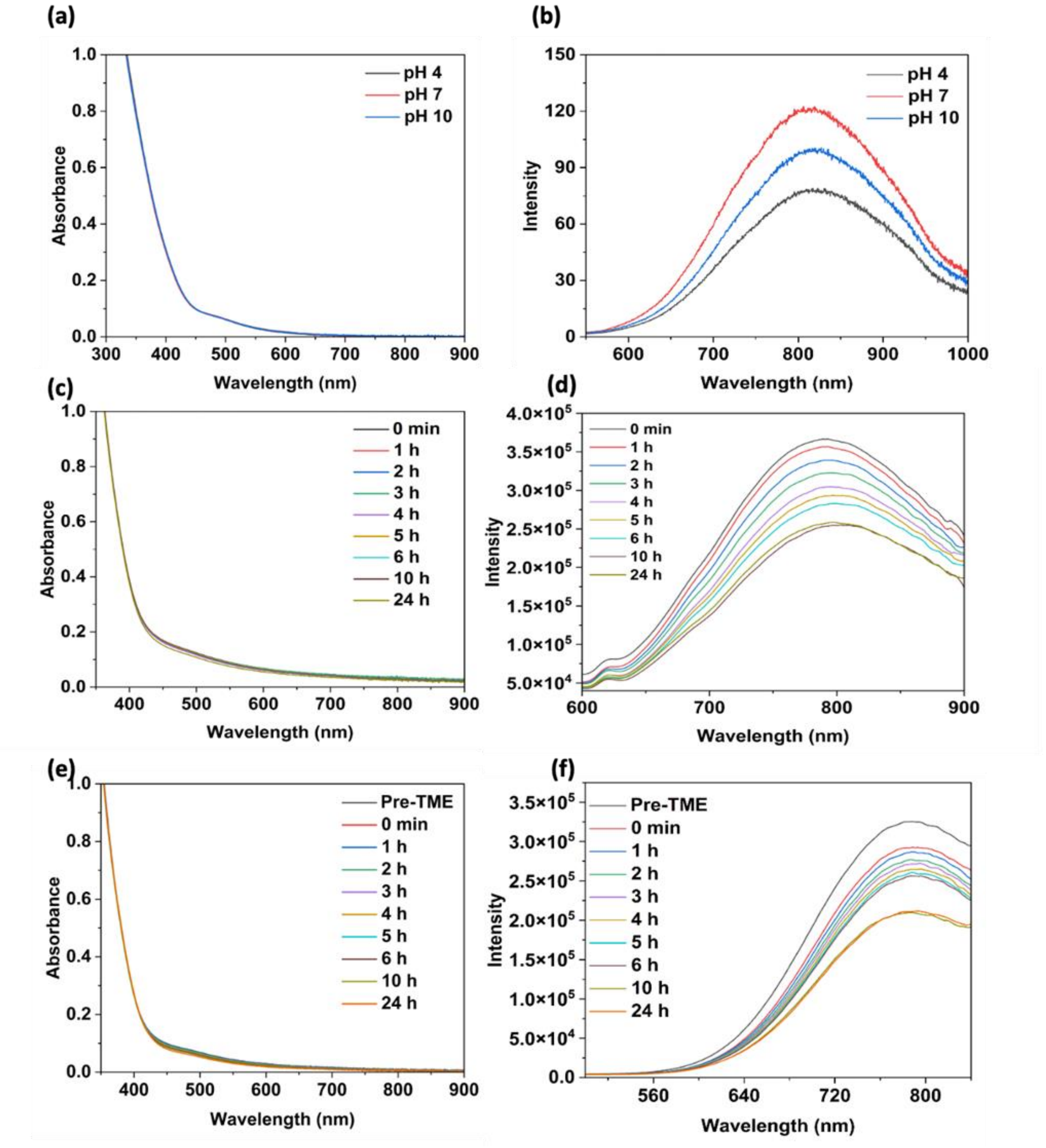

Figure S1. (a) absorbance and **(b)** emission spectra of Au_{22}(Lys-Cys-Lys)$_{16}$ nanoclusters at various pH. **(c)** absorbance and **(d)** emission spectra monitored for 24 h in 50% FBS in PBS. **(e,f)** corresponding spectra in TME conditions.

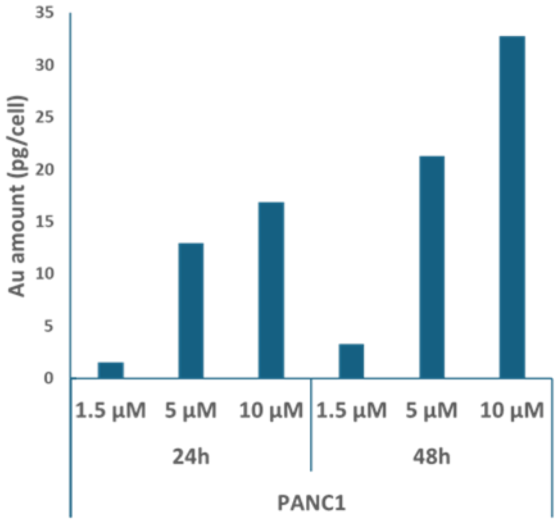

Figure S2. Nanocluster intracellular uptake (gold content measured by ICP-MS) in the PANC-1 cells, varying the incubation time and concentration.

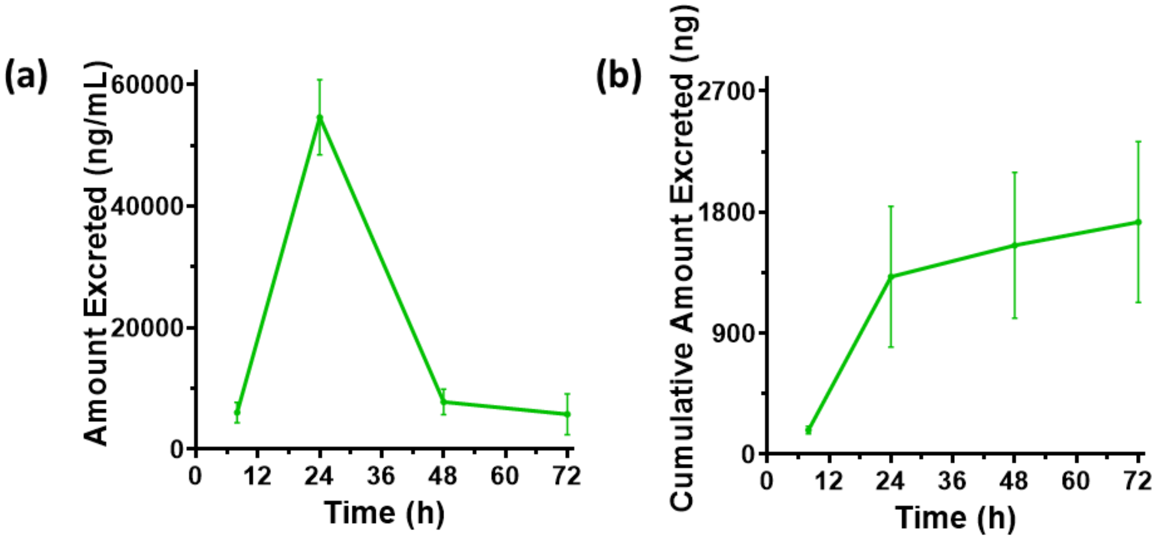

Figure S3. Renal clearance of AuNCs following 10 mg/kg i.v. injection: Means ± 1 s.d., N=5 mice **(a)** gold concentration in excreted urine at different time period, and **(b)** total gold in the collected urine.
